# A randomized pilot study of oncology massage to treat chemotherapy-induced peripheral neuropathy

**DOI:** 10.1038/s41598-022-23372-w

**Published:** 2022-11-08

**Authors:** Gabriel Lopez, Cathy Eng, Michael Overman, David Ramirez, Wenli Liu, Curtiss Beinhorn, Pamela Sumler, Sarah Prinsloo, Yisheng Li, Minxing Chen, Eduardo Bruera, Lorenzo Cohen

**Affiliations:** 1 Department of Palliative, Rehabilitation and Integrative Medicine, University of Texas, MD Anderson Cancer Center, 1515 Holcombe Blvd, Unit 1414, Houston, TX 77030 USA; 2 Division of Hematology and Oncology, Vanderbilt-Ingram Cancer Center, Nashville, TN USA; 3 Department of Gastrointestinal Medical Oncology, University of Texas, MD Anderson Cancer Center, Houston, TX USA; 4 Department of Breast Medical Oncology, University of Texas, MD Anderson Cancer Center, Houston, TX USA; 5 Department of Neurosurgery, University of Texas, MD Anderson Cancer Center, Houston, TX USA; 6 Department of Biostatistics, University of Texas, MD Anderson Cancer Center, Houston, TX USA

**Keywords:** Breast cancer, Gastrointestinal cancer, Chemotherapy, Quality of life, Palliative care, Adverse effects, Neurological manifestations, Chronic pain

## Abstract

This pilot randomized controlled trial investigated massage therapy for symptomatic relief of chemotherapy-induced peripheral neuropathy (CIPN) to determine the ideal weekly frequency and number of weeks of providing massage. We evaluated the feasibility and initial efficacy of a Swedish massage protocol to treat lower extremity (LE) CIPN. Inclusion criteria: LE neuropathy attributed to oxaliplatin, paclitaxel, or docetaxel, with no other attributable causes; ≥ 6 months since last chemotherapy; self-reported neuropathy score ≥ 3, 0–10 scale; age ≥ 18. Participant randomization (2:2:1:1) to one of four groups: LE (2) or head/neck/shoulder (control; 1) massage 3 times (3X) a week for 4 weeks; LE (2) or control (1) massage 2X/week for 6 weeks. Completion rate and the Pain Quality Assessment Scale (PQAS) was measured at baseline and 10 weeks later. 71 patients participated: 77.5% women; 57.7% (breast cancer), and 42.3% (GI cancer); mean age 60.3 y/o (range: 40–77); average > 3 years since last chemotherapy. Massage was deemed feasible: mean completion rates (max = 12) were 8.9 (SD 4.2) for 3X/week and 9.8 (SD 4.0) for 2X/week with no statistically significant differences. There were no statistically significant treatment group interactions in PQAS scores at 10-weeks follow-up. There was a statistically significant treatment schedule main effect for PQAS subscales (*p* < 0.05) at 10 weeks, with lower CIPN symptoms for 3X/week groups versus 2X/week groups. Improvements considered clinically significant favored the LE 3X/week group. Completion rates met pre-defined feasibility criteria. We seemed to observe better outcomes (CIPN symptom reduction) with the more intensive (3X/week for 4 weeks) massage intervention with no differences in adherence, regardless of whether the massage was directly to the CIPN-affected area or not. However, there was some suggestion that the massage program targeting the CIPN-affected area directly provided 3X a week for 4 weeks resulted in the best outcomes.

## Introduction

Each year thousands of cancer patients receive therapies that may contribute to chronic neurologic toxicity^1,2^. Such agents can include platinum compounds (cisplatin, oxaliplatin, carboplatin) and taxanes (paclitaxel, docetaxel). Oxaliplatin-based chemotherapy is commonly used for the treatment of colon cancer and other gastrointestinal malignancies; taxanes are commonly used for the treatment of breast malignancies. Up to half of these patients go on to develop chronic chemotherapy-induced peripheral neuropathy (CIPN)^3^.

CIPN is a primarily sensory, dose limiting toxicity that can have a detrimental impact on quality of life (QOL), decreasing physical functioning with debilitating symptoms including pain, numbness, tingling, parasthesias, and/or cold-sensitivity. The severity of neurotoxicity from chemotherapy is impacted by the specific agent used, the dose, the schedule of administration, and co-morbidities such as pre-existing neuropathic syndromes. Primary sites of chemotherapy-induced neurotoxicity are at the dorsal root ganglia for platinum agents and direct axonal toxicity for taxanes^2^. Both paclitaxel and docetaxel contribute to chronic persistent neurotoxicity involving the hands and/or feet. Lower extremities are the most commonly involved area in patients with chronic persistent oxaliplatin-induced peripheral neuropathy, with as many as 79% of patients with lower limb residual sensory neuropathy at a median follow-up of 25 months^3–5^. When comparing persistence of CIPN symptoms in patients who received oxaliplatin versus docetaxel at 1 year follow-up, individuals receiving both agents are more likely to have persistence of CIPN symptoms in the feet (63.% and 44.8%, respectively) versus hands (31.3% and 35.1%, respectively)^6^.

Despite ongoing research for the prevention and treatment of CIPN, there are no treatments accepted as the gold standard. A variety of pharmacologic treatments including glutamine, gabapentin, carbamazepine, vitamin E, and calcium/magnesium have been studied to prevent or alleviate CIPN symptoms. Only one pharmacologic agent, duloxetine, holds a moderate recommendation for treatment, not prevention, of CIPN^1^. Unfortunately, duloxetine has multiple unwanted side effects including nausea, weight loss, drowsiness, and dry mouth with modest benefits^7^.

Interest in and use of complementary and integrative medicine (CIM) approaches are increasing in western medical settings, with such modalities as massage showing promise in treating cancer- and cancer treatment-related symptoms^8^. Research on the benefits of massage for the treatment of peripheral neuropathy in diseases such as carpal tunnel syndrome and diabetes is suggestive^9,10^. In a single-arm study exploring the efficacy of massage for treating carpal tunnel syndrome, the treatment dose included a 30 min Swedish massage technique twice per week for 6 weeks, showing baseline to 6-weeks improvements, with proposed mechanisms including restoring neural conduction and nerve impairment. In a randomized controlled study of aromatherapy massage for neuropathic pain in patients with diabetes, massage was provided three times per week for 4 weeks, with significant reductions in neuropathic pain at the end of the study period, with proposed mechanisms including synergistic effects on analgesia, neuroprotection, sedation, and circulation. In the cancer setting, CIM treatments studied for CIPN include acupuncture and to a lesser extent massage^11,12^.

Massage encompasses a variety of techniques that involve direct manipulation of soft tissue^13^. Specialized training in oncology massage teaches therapists how to make treatment modifications including changes in position, special bolstering, adjustment of pressure and pace, and site restrictions. Oncology massage as a field has been exploring the use of specific massage protocols for treatment of CIPN. Our own pilot work (cohort-based pre/post assessments, case reports, and anecdotal experience) support the potential role of oncology massage in the management of CIPN^8^. To date, there is only one published case report of oncology massage for the treatment of CIPN^14^. There are no randomized trials of massage to treat CIPN and no information on the ideal frequency and dosing of oncology massage to treat CIPN.

The objectives of this pilot study were to explore the feasibility (primary aim) and initial efficacy (secondary aim) of two massage treatment schedules using a Swedish massage protocol accepted by oncology massage therapists for treatment of peripheral neuropathy^15^. We explored feasibility, patient adherence, effect size estimates, and initial efficacy of both treatment schedules in order to design a larger randomized clinical trial to evaluate the efficacy of massage for the treatment of CIPN.

## Methods

This single-blind (patients blinded), pilot study was designed to evaluate the optimum treatment schedule and initial efficacy of two treatment schedules of a standardized Swedish massage technique to treat chronic, lower extremity (LE) CIPN. The study was approved by the MD Anderson Cancer Center institutional review board (ClinicalTrials.gov Identifier: NCT02221700, registered 20/08/2014; IRB Protocol # 2014-0250). All research was performed in accordance with relevant guidelines/regulations including with the Declaration of Helsinki; informed consent was obtained from all participants.

Study participants were identified from oncology clinical centers through distribution of electronic and paper fliers, or via direct mail. Inclusion criteria were neuropathy attributed to oxaliplatin, paclitaxel, or docetaxel, with no prior history of attributable causes for CIPN; self-reported neuropathy score ≥ 3 on 0–10 scale and/or grade 2 or 3 neuropathy according to NCI CTC criteria; greater or equal to 6 months since last chemotherapy treatment; and age > 18. The self-reported neuropathy score is assessed as part of the standard of care in our integrative medicine center together with other symptoms that are part of the Edmonton Symptom Assessment Scale (ESAS); patients are asked to rate their neuropathy on a scale of 0–10, 10 being the worst, over the prior 24-h period. Participants must have been on a stable dose of medications for CIPN symptom management within 2 weeks of study enrollment, all drug classes allowed including duloxetine and pregabalin; stable dose was defined as: (1) no change in drug class; (2) increases or decreases that are less than or equal to 20% of the total dosage. Participants were taken off study if they changed medication (drug class) for pain control.

Exclusion criteria were: peripheral neuropathy pre-dating their chemotherapy; platelets < 50,000 or absolute neutrophil count < 500 within 6 months of enrollment; deep venous thrombosis (DVT) diagnosed within 12 months of enrollment or history of untreated LE DVT; bone metastases; active skin infection; lymphedema involving the treatment field; positive urine pregnancy test; or diagnosis of diabetes.

The recruitment goal was 90 participants. In enrolling 30 patients per treatment group, we expected a drop out of 20% with a final enrollment of 24 patients per treatment group. Once baseline measures were collected, participants were randomized to one of four massage groups in a 2:2:1:1 randomization ratio (Table 1). The alternate-site massage groups were created to control for symptom change over time and treatment effect (therapeutic presence) of the massage therapist. Measures in the control groups were collected at comparable time points as the other groups. Randomization was conducted using minimization based on age, sex, stage of disease, type of disease (breast or gastrointestinal), degree of neuropathy [as defined by baseline Pain Quality Assessment Scale (PQAS) total score], and time since diagnosis^16^.

**Table 1:** Study group assignments.

*Group 1*: primary intervention group (site-specific lower extremity massage); massage schedule: three times (3X) a week for 4 weeks; n = 30
*Group 2*: primary intervention group (site-specific lower extremity massage); massage schedule: two times (2X) a week for 6 weeks; n = 30
*Groups 3 and 4*: control groups (head/neck/shoulder, alternate-site massage); participants (n = 15) assigned to each group mirroring the massage schedules of the two primary intervention groups (3X and 2X)

### Intervention

Massage treatments were provided by one of three licensed oncology massage therapists, each with > 15 years experience. For the site-specific (LE) massage Group 1 and Group 2, massage practice followed a standardized protocol for CIPN. The total visit time was ≤ 30 min, including preparation, positioning, and 10–12.5 min of massage per leg. The setting had a controlled temperature with no background music. Patients were in the semi-Fowler position with knees bolstered. A hypoallergenic, unscented lotion was applied to both LE below the knees. The massage technique started distally in the toes, ending at the knee. Massage pressure was gradually increased, per patient tolerance, following oncology massage guidelines.

For the alternate-site massage control groups (Groups 3 and 4), massage practice involved the scalp/neck/shoulders and back above T4. For participants at risk for secondary lymphedema of the upper quadrant or other unique health conditions, positioning and massage was modified according to oncology massage safety standards. For treatment fidelity, a video and printed copy of the massage treatment protocol were available for review.

### Assessments

In addition to comparison of completion rates of 2X versus 3X a week as part of the primary aim, participant assessment included demographic information and a self-reported measure assessing neuropathic pain. We assessed pretreatment expectations and conducted exit interviews for all participants. To be evaluable, assessments must have taken place within a ± 7 days window of the scheduled time. Assessment measure responses were collected either electronically or by pen and paper and stored in a secure FileMaker Pro database. Data was collected at baseline, midpoint, and at 10 weeks after baseline (end-of-study).

For the purpose of this study, we focused on the 10-weeks follow-up to determine any lasting effects from the massage. Time from baseline to the 10-weeks end-of-treatment follow-up was consistent across massage treatment schedule groups to control for improvements that could take place simply due to the passage of time. This meant that there was a difference in time from end of last massage to 10-weeks follow-up depending on treatment schedule group (4 weeks from last massage for the 2X per week groups and 6 weeks for the 3X per week groups).

*Pain Quality Assessment Scale (PQAS)* was the primary outcome measure for the secondary aim. PQAS is a 20-item measure developed to quantify quality and intensity of neuropathic symptoms^17^. It was derived from the Neuropathic Pain Scale and includes symptom descriptors common to people experiencing neuropathic pain or other neuropathic sequelae. Subscales of the PQAS include: Paroxysmal Pain (PP; shooting, sharp, electric, hot, and radiating); Superficial Pain (SP; itchy, cold, numb, sensitive, and tingling); and Deep Pain (DP; aching, heavy, dull, cramping, and throbbing). Secondary analyses included examining the individual items and subscale scores where a 2-point change or greater was considered clinically significant^18^.

*Expectation assessments* were on a 5-point scale (not at all agree, a little agree, moderately agree, mostly agree, completely agree) and asked about expectation of massage effects on CIPN.

### Statistical analysis

Assuming a 20% drop out rate, the sample size used to assess the primary aim was 30 per arm. This sample size provides 80% power to detect a standardized difference of 0.736 SD units between the two treatment arms at a two-sided 5% significance level.

Data was summarized by descriptive statistics including mean and standard deviation, median and range for continuous variables and frequency and proportion for categorical variables. For the primary aim, the completion rate was defined as the average number of treatments completed of a possible 12 for patients in each treatment group. Optimum treatment schedule in terms of adherence was determined by using a Wilcoxon rank-sum test comparing number of massages completed between the two possible treatment schedules. For the secondary aim, we examined the effects of location (LE vs control), schedule (2X vs 3X/week) and their interaction on the PQAS subscales of PQAS-SP, PQAS-DP, and PQAS-PP at 10 weeks (end-of-study) using the Ordinary Least Squares (OLS) method for linear regression controlling for baseline levels. Multilevel linear modelling with random intercepts was used to assess location by schedule, location by time (visits 6, 12, and week 10), schedule by time interaction effects, and main effects on the PQAS subscales, controlling for the baseline outcome. Visits 6 and 12 correspond to weeks 3 and 6 for the 2X/week schedule groups, and weeks 2 and 4 for the 3X/week schedule groups, respectively. For individual PQAS items, we descriptively reported change score based on group assignment. Wilcoxon rank-sum test was applied to evaluate the association between each factor (location or schedule) with responses.

### Ethics approval

The study was approved by the MD Anderson Cancer Center institutional review board (ClinicalTrials.gov Identifier: NCT02221700; IRB Protocol # 2014-0250). No separate ethics approval was required.

### Consent to participate

Informed consent was obtained from all individual participants included in the study. The study was approved by the MD Anderson Cancer Center institutional review board (ClinicalTrials.gov Identifier: NCT02221700, registered 20/08/2014; IRB Protocol # 2014-0250). All research was performed in accordance with relevant guidelines/regulations including with the Declaration of Helsinki.

## Results

**Figure 1:**
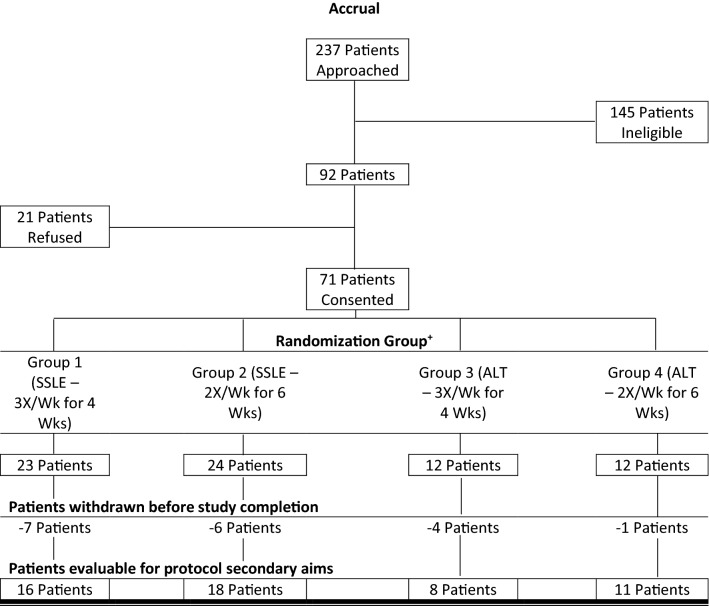
Oncology massage CIPN pilot consort diagram. +All patients randomized were considered evaluable for protocol primary aim. SSLE: Site specific lower extremity (primary intervention group). ALT: Alternate treatment site (head/neck/shoulder, control group).

Out of 237 patients approached (9/30/2015–1/08/2018), 92 were eligible, with 71 enrolled (Fig. 1, consort diagram). Of those enrolled, 77.5% were women, 57.7% with a diagnosis of breast cancer and 42.3% with a GI malignancy, mean age of 60.3, on average > 3 years (0.5–12; SD 2.6) from their last chemotherapy exposure (see Table 2). There were no group differences on demographic or medical variables.

**Table 2:** Demographics and baseline characteristics.

Age at consent	Mean ± Std (n)	60.8 ± 9.8 (71)
	Median (Min, Max)	61.0 (40.0, 77.0)
Gender	Female	55 (77.5%)
	Male	16 (22.5%)
Ethnicity	Not Hispanic or Latino	61 (88.4%)
	Hispanic or Latino	8 (11.6%)
Race	Black/African American	10 (14.1%)
	White/Caucasian	56 (78.9%)
	Asian/Oriental/Pacific Islander	1 (1.4%)
	Other	4 (5.6%)
Marital status	Married	47 (66.2%)
	Widowed	5 (7.0%)
	Divorced	15 (21.1%)
	Other	4 (5.6%)
Education Level	High school graduate/GED	3 (4.3%)
	Technical school or some college after high school	12 (17.2%)
	Associates degree (2 years degree)	10 (14.3%)
	College graduate (4-years degree)	18 (25.7%)
	Some graduate/professional school after college	10 (14.3%)
	Graduate or professional degree after college	17 (24.3%)
Employment	Yes, full-time or part-time	31 (45.6%)
	Yes, on disability or off work for treatment	4 (5.8%)
	No, retired or other	31 (48.5%)
Income	less than $20,000	4 (6.4%)
	$20,001–$50,000	16 (25.4%)
	$50,001–$75,000	8 (12.7%)
	More than $75,001	35 (55.5%)
Religious	Catholic	15 (21.7%)
	Protestant	26 (37.7%)
	Jewish	3 (4.3%)
	Other	25 (36.2%)
Cancer type	Breast	41 (57.7%)
	GI	30 (42.3%)
Cancer stage	I	9 (12.7%)
	II	24 (33.8%)
	III	30 (42.3%)
	IV	8 (11.3%)

### Completion rate

The mean completion rate was 8.94 (SD 4.24) massage treatments out of 12 for the 3X per week protocol and 9.83 (SD 4.04) out of 12 for the 2X per week protocol. Completion rates for individual treatment groups included: Group 1 mean 9.04 massages (SD 4.4, n = 23); Group 2 mean 9.5 massages (SD 4.4, n = 24); Group 3 mean 8.75 massages (SD 4.0, n = 12); and Group 4 10.5 massages (SD 3.4, n = 12). There was no statistically significant difference in completion rate between the 3X per week versus 2X per week massage protocols, collapsed by treatment location (*p* = 0.301, Wilcoxon rank-sum test; *p *= 0.368, t-test). Regression analysis taking into account treatment schedule and location of massage (LE or control) did not change the completion rate outcomes.

### PQAS subscale analysis

We obtained frequency and summary statistics for each treatment group at baseline and end-of-study (Table 3). Using OLS regression controlling for baseline symptom burden revealed no statistically significant differences between the LE massage treatment groups (Groups 1 + 2) and head/neck/shoulder control groups (Groups 3 + 4) for all three PQAS subscales at the 10-weeks follow-up (PQAS_DP *p* = 0.453; PQAS_PP *p* = 0.26; PQAS_SP *p* = 0.11) (Table 4). However, there was a main effect for treatment schedule (PQAS_DP *p* = 0.008; PQAS_PP *p* = 0.025; PQAS_SP *p* = 0.001) with participants getting massage 3X week (Group 1 + Group 3) reporting significantly lower (better) scores on all three PQAS outcomes than those getting massage 2X week (Group 2 + Group 4) (see Tables 3 and 4). Multilevel linear modeling for all three PQAS subscales revealed no statistically significant location (LE vs. control) by schedule, location by time (visits 6, 12 and week 10), or schedule by time interaction effects (*p* > 0.05). However, like the above analyses, there was a statistically significant treatment schedule main effect, collapsed over time, for PQAS subscales DP (*p* = 0.032) and SP (*p* = 0.001), with lower (better) scores for 3X/week groups than 2X/week groups, regardless of follow-up time point. In contrast, there was no statistically significant schedule main effect over time for PQAS subscale PP (*p* = 0.153). There was a statistically significant treatment location main effect, collapsed over time, for PQAS subscale PP (*p* = 0.041), favoring LE over control; the same was not true for the location main effect for DP (*p* = 0.325) or SP (*p* = 0.202).

**Table 3:** PQAS subscale summary statistics by treatment arms and time points (baseline and week 10).

Treatment group	Baseline mean (SD)	Week 10, end of study mean (SD)
**A: PQAS_DP (deep pain)**		
Group 1 (LE 3X)	4.07 (2.01) n = 23	1.75 (1.8) n = 16
Group 2 (LE 2X)	4.38 (2.76) n = 23	3.58 (1.7) n = 16
Group 3 (HN 3X)	3.64 (2.02) n = 11	1.97 (1.05) n = 6
Group 4 (HN 2X)	5.25 (2.93) n = 12	4.12 (3.09) n = 10
	p* = 0.425	*p* = 0.015
Group 1 + Group 3 (3X)	3.93 (1.99) n = 34	1.81 (1.61) n = 22
Group 2 + Group 4 (2X)	4.68 (2.81) n = 35	3.78 (2.29) n = 26
	*p* = 0.206	*p* = 0.001
Group 1 + Group 2 (LE)	4.23 (2.39) n = 46	2.66 (1.96) n = 32
Group 3 + Group 4 (HN)	4.48 (2.61) n = 23	3.31 (2.69) n = 16
	*p* = 0.690	*p* = 0.345
**B. PQAS_PP (paroxysmal pain)**		
Group 1 (LE 3X)	3.74 (2.25) n = 23	1.26 (1.79) n = 16
Group 2 (LE 2X)	3.97 (2.47) n = 24	2.94 (2.22) n = 17
Group 3 (HN 3X)	3.07 (2.52) n = 11	1.23 (1.64) n = 6
Group 4 (HN 2X)	4.48 (2.93) n = 12	3.42 (2.68) n = 10
	*p* = 0.587	*p* = 0.032
Group 1 + Group 3 (3X)	3.52 (2.32) n = 34	1.25 (1.71) n = 22
Group 2 + Group 4 (2X)	4.14 (2.6) n = 36	3.12 (2.36) n = 27
	*p* = 0.301	*p* = 0.003
Group 1 + Group 2 (LE)	3.86 (2.34) n = 47	2.13 (2.16) n = 33
Group 3 + Group 4 (HN)	3.81 (2.77) n = 23	2.6 (2.53) n = 16
	*p* = 0.942	*p* = 0.501
**C. PQAS_SP (surface pain)**		
Group 1 (LE 3X)	4.43 (1.98) n = 23	2.17 (1.98) n = 16
Group 2 (LE 2X)	4.47 (2) n = 24	3.72 (1.93) n = 17
Group 3 (HN 3X)	4.65 (1.71) n = 12	2.4 (1.39) n = 6
Group 4 (HN 2X)	4.85 (2.54) n = 11	4.6 (2.85) n = 9
	*p* = 0.942	*p* = 0.031
Group 1 + Group 3 (3X)	4.5 (1.87) n = 35	2.24 (1.81) n = 22
Group 2 + Group 4 (2X)	4.59 (2.15) n = 35	4.02 (2.28) n = 26
	*p* = 0.850	*p* = 0.005
Group 1 + Group 2 (LE)	4.45 (1.97) n = 47	2.97 (2.08) n = 33
Group 3 + Group 4 (HN)	4.75 (2.1) n = 23	3.72 (2.57) n = 15
	*p* = 0.564	*p* = 0.288

*LE* lower extremity treatment arm, *HN* head neck shoulder control arm, *2X* two times per week massage treatment, *3X* three times per week massage treatment.

p*: ANOVA *p*-value.

**Table 4:** Ordinary least squares (OLS) regression results for PQAS subscales (secondary outcomes) at Week 10 (end of study).

Variable	Model			
	Estimate	SE	95% CI	*p*-value
**A PQAS_DP (deep pain; n = 48)**				
Schedule^a^	− 1.35	.48	(− 2.32, − .38)	.008
Location^b^	− .38	.50	(− 1.37, .62)	.453
**B PQAS_PP (paroxysmal pain; n = 49)**				
Schedule^a^	− 1.19	.51	(− 2.23, − .16)	.025
Location^b^	− .61	.53	(− 1.68, .46)	.26
**C PQAS_SP (surface pain; n = 47)**				
Schedule^a^	− 1.68	.49	(− 2.66, − .7)	.001
Location^b^	− .87	.53	(− 1.93, .2)	.108

^a^2X per week (Group 2 + Group 4) vs 3X per week (Group 1 + Group 3); 2X per week used as reference.

^b^Lower extremity (Group 1 + Group 2, intervention) versus head/neck/shoulder massage (Group 3 + Group 4, control); head/neck/shoulder used as reference.

### PQAS individual item analysis

Examining mean change of individual PQAS items between treatment groups, Group 1 (LE, 3X per week) had consistently greater mean score reduction across multiple PQAS items than the other three treatment groups, although these comparisons were not statistically significant (Table 5). For Group 1, there was a > 2 point improvement in 16 out of 20 PQAS individual items with 6 items greater than a 3 point reduction; Group 2 had no items with > 2 point improvement; Group 3 had only two items with > 2 point improvement; and Group 4 had five items with > 2 point improvement. For example, for PQAS item 1 (intense pain sensation), Group 1 mean score reduction (improvement) was 3.1 versus 1.4 for Group 2, 0.8 for Group 3, and 1.9 for Group 4. This suggests a clinically significant improvement (2 + reduction) for Group 1 versus the other groups on at least 10 of the 20 items. Comparing mean differences between baseline and end of study for the four groups, *p*-values were significant (*p* < 0.05) for PQAS items 6, 7, and 14.

**Table 5:** Individual PQAS changes scores [End-of-Study (week 10) minus Baseline].

PQAS items		Group 1 LE	Group 2 LE	Group 3 HNS	Group 4 HNS	ANOVA *p*-value
PQAS 1—intense	Mean ± SD (n)	**− 3.1 ± 2.8 (16)**	− 1.4 ± 1.8 (17)	− 0.8 ± 3.7 (6)	− 1.4 ± 2.2 (10)	0.126
PQAS 2—sharp	Mean ± SD (n)	**− 3.4 ± 3.2 (16)**	− 1.8 ± 2.6 (17)	− 0.3 ± 2.7 (6)	− 1.3 ± 2.3 (10)	0.088
PQAS 3—hot	Mean ± SD (n)	− 1.7 ± 2.9 (16)	− 1.4 ± 2.7 (17)	− 1.5 ± 1.8 (6)	**− 2.0 ± 1.9 (10)**	0.947
PQAS 4—dull	Mean ± SD (n)	**− 3.0 ± 3.5 (16)**	− 0.9 ± 3.3 (17)	0.2 ± 4.6 (6)	− 0.5 ± 3.0 (10)	0.152
PQAS 5—cold	Mean ± SD (n)	**− 2.2 ± 2.3 (16)**	− 0.8 ± 4.1 (17)	− 1.3 ± 1.8 (6)	− 0.2 ± 1.5 (9)	0.377
PQAS 6—sensitive	Mean ± SD (n)	**− 2.9 ± 2.7 (16)**	− 0.1 ± 3.2 (17)	0.0 ± 3.8 (6)	1.0 ± 2.4 (9)	0.010
PQAS 7—tender	Mean ± SD (n)	**− 3.1 ± 3.1 (16)**	− 1.4 ± 1.9 (17)	− 0.8 ± 3.5 (6)	− 0.3 ± 1.6 (10)	0.040
PQAS 8—itchy	Mean ± SD (n)	− 1.7 ± 2.4 (16)	− 0.1 ± 2.9 (17)	− 0.2 ± 1.0 (6)	− 0.4 ± 1.8 (10)	0.254
PQAS 9—shooting	Mean ± SD (n)	**− 2.9 ± 2.3 (16)**	− 1.6 ± 3.6 (17)	0.2 ± 2.0 (6)	− 0.8 ± 1.3 (10)	0.072
PQAS 10—numb	Mean ± SD (n)	**− 2.8 ± 3.0 (16)**	− 1.5 ± 2.9 (17)	**− 2.0 ± 2.4 (6)**	− 1.7 ± 2.0 (10)	0.549
PQAS 11—electrical	Mean ± SD (n)	**− 2.7 ± 2.5 (16)**	0.1 ± 3.8 (17)	0.3 ± 4.2 (6)	**− 2.0 ± 2.2 (10)**	0.057
PQAS 12—tingling	Mean ± SD (n)	**− 3.4 ± 2.7 (16)**	− 1.2 ± 2.1 (17)	− 1.8 ± 1.9 (6)	**− 2.1 ± 2.5 (10)**	0.081
PQAS 13—cramping	Mean ± SD (n)	**− 2.4 ± 2.3 (16)**	− 1.1 ± 3.1 (17)	**− 3.7 ± 3.0 (6)**	− 0.5 ± 2.1 (10)	0.076
PQAS 14—radiating	Mean ± SD (n)	**− 2.3 ± 2.8 (16)**	− 1.0 ± 2.9 (17)	1.7 ± 2.9 (6)	− 0.9 ± 1.9 (10)	0.033
PQAS 15—throbbing	Mean ± SD (n)	− 1.9 ± 2.6 (16)	− 1.2 ± 3.1 (16)	− 1.2 ± 2.7 (6)	− 0.4 ± 1.5 (10)	0.583
PQAS 16—aching	Mean ± SD (n)	**− 2.3 ± 3.1 (16)**	− 0.7 ± 2.9 (17)	− 1.0 ± 3.0 (6)	**− 2.0 ± 1.8 (10)**	0.392
PQAS 17—heavy	Mean ± SD (n)	− 1.1 ± 1.5 (16)	− 1.1 ± 2.6 (17)	− 0.2 ± 3.3 (6)	**− 2.2 ± 2.7 (10)**	0.418
PQAS 18—unpleasant	Mean ± SD (n)	**− 3.2 ± 3.1 (16)**	− 1.5 ± 2.0 (17)	− 1.7 ± 2.3 (6)	− 1.9 ± 2.3 (10)	0.265
PQAS 19a—intense deep	Mean ± SD (n)	**− 2.7 ± 3.4 (16)**	− 1.1 ± 2.7 (15)	− 0.7 ± 3.0 (6)	− 1.4 ± 2.4 (9)	0.385
PQAS 19b—intense surface	Mean ± SD (n)	−** 2.3 ± 2.5 (16)**	− 0.9 ± 2.3 (15)	− 0.7 ± 5.0 (6)	− 0.7 ± 3.1 (9)	0.476
PQAS 20—time quality (intermittent, variable, stable)	Mean ± SD (n)	0.1 ± 0.6 (15)	0.1 ± 0.4 (15)	− 0.6 ± 0.9 (5)	− 0.1 ± 0.6 (9)	0.090

Group 1: intervention (lower extremity, LE) massage 3 times per week for 4 weeks.

Group 2: intervention (lower extremity, LE) massage 2 times per week for 6 weeks.

Group 3: control (head/neck/shoulder, HNS) massage 3 times per week for 4 weeks.

Group 4: control (head/neck/shoulder, HNS) massage 2 times per week for 6 weeks.

Bold represents > 2-point change (improvement).

### Expectancy scale

We also explored the association between patient baseline expectancy and the PQAS individual item outcomes. The baseline expectancy scale was added to the logistic regression model of PQAS at week 10 to evaluate whether there was any association between expectancy and the PQAS outcomes; however, the results indicated no statistically significant associations, controlling for treatment schedule and massage location (i.e., for fixed treatment schedule and massage location).

### Exit interview

On the exit interview assessment, the majority of participants agreed that their neuropathy improved (88.2%), ability to cope with their neuropathy improved (86%), and felt more confident with their daily activities (88%). The majority “mostly” or “completely” agreed that their massage schedule was acceptable (84.3%) and recommended others receive massage treatment for CIPN management (80.4%). There were no obvious differences in the satisfaction or preference outcomes between groups.

## Discussion

Our pilot study explored the feasibility and initial efficacy of two different oncology massage delivery protocols provided either two times a week (2X) for 6 weeks or three times (3X) a week for 4 weeks. The study also examined the differences between delivering the massage to the CIPN affected area (LE) or to a control site (head/neck/shoulder area). In designing a future trial, we wanted to determine if greater frequency of treatment (3X compared to 2X) over fewer weeks (4 weeks versus 6 weeks) would result in worse completion rate due to additional burden of more frequent visits for the massage treatment. We succeeded in demonstrating feasibility of both treatment schedules, with no statistically significant differences observed in completion rates between the 2X and 3X treatment schedules.

Examination of initial efficacy based on the PQAS subscales revealed that there was no difference in those who received massage to an area affected by CIPN or to an alternate, unaffected site. On review of pre/post massage treatment assessments (standard-of-care), our own earlier experience with outpatient oncology massage (treatment plan based on presenting symptoms, not protocolized) suggests clinically significant benefit of massage for neuropathy independent of treatment site^8^. However, the 3X treatment groups, regardless of massage treatment location, had clinically and statistically significant better relief of symptoms than the 2X treatment groups. Dose-density was more important than location when examining the three PQAS subscales. Other studies of massage have explored the importance of dose when it comes to outcomes^9,10,19^. Although our study recruitment enrolled participants based on the presence of LE neuropathy and the primary intervention was the LE massage protocol, overall improvement in neuropathy was associated the increased frequency of massage treatment (3X vs 2X), rather than massage treatment location. Such an observation that massage to the head/neck/shoulder area contributed to improvement in LE neuropathic discomfort may suggest that the benefits of massage treatment for neuropathy are in part due to systemic effects, rather than limited to local effects. However, examination of individual PQAS variables (Table 5) revealed clinically significant mean improvement (> 2 point reduction)^18^ in multiple symptoms that favored the 3X schedule group receiving targeted massage to the affected area with changes and differences reaching clinical significance in 16 of 20 symptoms.

We also observed statistically significant sustained improvement in symptom score reduction at 6 weeks after completion of the last massage treatment in the 3X massage group (group where all massages were delivered within a 4-weeks period). Because time from baseline to end-of-study was the same for all groups, the 3X group (4-weeks treatment window) was actually 2 more weeks out from their last massage than the 2X group (6-weeks treatment window), suggesting an even greater sustained effect of the more dose-dense massage treatment as the effects of the massage are expected to diminish the further out you get from the last massage treatment. Prior massage therapy research for different symptoms suggests that time from last massage is associated with the waning of the effects^20^. Another earlier study in healthy adults exploring the biological effects of Swedish massage over a 5 weeks period suggests the presence of cumulative and sustained effects of massage as dependent on dose of massage delivered. They observed greater biological change with higher doses, twice versus once per week. Our finding that the effects of the massage may persist weeks beyond the actual hands-on intervention opens the question as to whether or not there may be a role for maintenance massage treatments to help further sustain the observed effects.

Limitations of the study include that it was conducted at a single academic center, which may not represent patients receiving community cancer care. Recruiting participants with gastrointestinal and breast cancers and neuropathy secondary to oxaliplatin, paclitaxel, and docetaxel, our observations regarding feasibility and efficacy may not represent findings with other malignancies or neurotoxic agents. An additional limitation is that we did not have a usual care or placebo control groups—all of our participants received massage, whether in the primary treatment arm or active control group. Without a control or usual care (wait list) group, it is more difficult to account for natural resolution of CIPN over time. However, not having a control group does not affect our ability to compare treatment arms. Of note, it is unique to include a comparison massage treatment group in this type of research. Although the massage dose was delivered over either a 4- or 6-weeks period depending on the group assignment, comparisons were made for both groups at the 10-weeks time point from baseline to reduce possible confounding effects of natural CIPN resolution over time. However, the 2-weeks difference between groups from last massage to the 10-weeks assessment is a confounder. Yet the majority of our participants were more than 3 years out from their neurotoxic chemotherapy exposure, suggesting that natural resolution of neuropathy during the short study period would be less likely. We also did not find any group by time effects for massage dose. Another limitation includes not surveying participants beyond the 10-weeks study period, making it difficult to draw conclusions about the durability of the observed response and/or the potential role for maintenance massage treatment. Finally, due to the feasibility nature of the study, no multiple testing adjustments are made in our statistical analyses, suggesting that all results should be taken as for hypothesis generation.

It is important to note a scale such as the PQAS, although useful in quantifying the quality and intensity of neuropathic symptoms, does not capture aspects of how the neuropathic symptoms interfere with function. Our current study explores feasibility and initial efficacy of our massage protocol for treating CIPN; for future studies, it will be important to include aspects of function which can be captured more comprehensively by tools such as the FACT/GOG-NTX-13^21^, EORTC-QLQ-CIPN20^22^, and PRO-CTCAE^23^.

## Conclusion

Our study demonstrated high feasibility of providing two different oncology massage programs and schedules to cancer survivors affected by CIPN. Providing massage 3X a week for 4 weeks seemed to result in better outcomes than 2X a week for 6 weeks, with no differences in adherence. There was also some suggestion that the massage program targeting the CIPN-affected area directly provided 3X a week for 4 weeks resulted in the best outcomes. Preliminary data from this pilot study will inform the choice of the ideal schedule in order to conduct a larger randomized clinical trial that would include a proper placebo control group. Mounting evidence can help support efforts at increasing patient access to oncology massage treatments for symptom relief. Current challenges including variable levels of insurance coverage and limited access to providers with specialized oncology massage training.

## Data Availability

The datasets used and analyzed during the current study are available from the corresponding author on reasonable request.

## Code availability

There is no code associated with this manuscript and/or research study.
