# Publisher Correction: A randomized pilot study of oncology massage to treat chemotherapy-induced peripheral neuropathy

**DOI:** 10.1038/s41598-023-34877-3

**Published:** 2023-05-23

**Authors:** Gabriel Lopez, Cathy Eng, Michael Overman, David Ramirez, Wenli Liu, Curtiss Beinhorn, Pamela Sumler, Sarah Prinsloo, Yisheng Li, Minxing Chen, Eduardo Bruera, Lorenzo Cohen

**Affiliations:** 1 Department of Palliative, Rehabilitation and Integrative Medicine, University of Texas, MD Anderson Cancer Center, 1515 Holcombe Blvd, Unit 1414, Houston, TX 77030 USA; 2 Division of Hematology and Oncology, Vanderbilt-Ingram Cancer Center, Nashville, TN USA; 3 Department of Gastrointestinal Medical Oncology, University of Texas, MD Anderson Cancer Center, Houston, TX USA; 4 Department of Breast Medical Oncology, University of Texas, MD Anderson Cancer Center, Houston, TX USA; 5 Department of Neurosurgery, University of Texas, MD Anderson Cancer Center, Houston, TX USA; 6 Department of Biostatistics, University of Texas, MD Anderson Cancer Center, Houston, TX USA

Correction to: *Scientific Reports* 10.1038/s41598-022-23372-w, published online 08 November 2022

The original version of this Article contained errors in Table 5, where the headings of the columns were incorrectly formatted. The correct and incorrect Table appear below.

Correct:

**Table 5:** Individual PQAS changes scores [End-of-Study (week 10) minus Baseline].

PQAS items		Group 1 LE	Group 2 LE	Group 3 HNS	Group 4 HNS	ANOVA p-value
PQAS 1—intense	Mean ± SD (n)	**− 3.1 ± 2.8 (16)**	− 1.4 ± 1.8 (17)	− 0.8 ± 3.7 (6)	− 1.4 ± 2.2 (10)	0.126
PQAS 2—sharp	Mean ± SD (n)	**− 3.4 ± 3.2 (16)**	− 1.8 ± 2.6 (17)	− 0.3 ± 2.7 (6)	− 1.3 ± 2.3 (10)	0.088
PQAS 3—hot	Mean ± SD (n)	− 1.7 ± 2.9 (16)	− 1.4 ± 2.7 (17)	− 1.5 ± 1.8 (6)	**− 2.0 ± 1.9 (10)**	0.947
PQAS 4—dull	Mean ± SD (n)	**− 3.0 ± 3.5 (16)**	− 0.9 ± 3.3 (17)	0.2 ± 4.6 (6)	− 0.5 ± 3.0 (10)	0.152
PQAS 5—cold	Mean ± SD (n)	**− 2.2 ± 2.3 (16)**	− 0.8 ± 4.1 (17)	− 1.3 ± 1.8 (6)	− 0.2 ± 1.5 (9)	0.377
PQAS 6—sensitive	Mean ± SD (n)	**− 2.9 ± 2.7 (16)**	− 0.1 ± 3.2 (17)	0.0 ± 3.8 (6)	1.0 ± 2.4 (9)	0.010
PQAS 7—tender	Mean ± SD (n)	**− 3.1 ± 3.1 (16)**	− 1.4 ± 1.9 (17)	− 0.8 ± 3.5 (6)	− 0.3 ± 1.6 (10)	0.040
PQAS 8—itchy	Mean ± SD (n)	− 1.7 ± 2.4 (16)	− 0.1 ± 2.9 (17)	− 0.2 ± 1.0 (6)	− 0.4 ± 1.8 (10)	0.254
PQAS 9—shooting	Mean ± SD (n)	**− 2.9 ± 2.3 (16)**	− 1.6 ± 3.6 (17)	0.2 ± 2.0 (6)	− 0.8 ± 1.3 (10)	0.072
PQAS 10—numb	Mean ± SD (n)	**− 2.8 ± 3.0 (16)**	− 1.5 ± 2.9 (17)	**− 2.0 ± 2.4 (6)**	− 1.7 ± 2.0 (10)	0.549
PQAS 11—electrical	Mean ± SD (n)	**− 2.7 ± 2.5 (16)**	0.1 ± 3.8 (17)	0.3 ± 4.2 (6)	**− 2.0 ± 2.2 (10)**	0.057
PQAS 12—tingling	Mean ± SD (n)	**− 3.4 ± 2.7 (16)**	− 1.2 ± 2.1 (17)	**− **1.8 ± 1.9 (6)	**− 2.1 ± 2.5 (10)**	0.081
PQAS 13—cramping	Mean ± SD (n)	**− 2.4 ± 2.3 (16)**	**− **1.1 ± 3.1 (17)	**− 3.7 ± 3.0 (6)**	**− **0.5 ± 2.1 (10)	0.076
PQAS 14—radiating	Mean ± SD (n)	**− 2.3 ± 2.8 (16)**	− 1.0 ± 2.9 (17)	1.7 ± 2.9 (6)	− 0.9 ± 1.9 (10)	0.033
PQAS 15—throbbing	Mean ± SD (n)	− 1.9 ± 2.6 (16)	− 1.2 ± 3.1 (16)	− 1.2 ± 2.7 (6)	− 0.4 ± 1.5 (10)	0.583
PQAS 16—aching	Mean ± SD (n)	**− 2.3 ± 3.1 (16)**	− 0.7 ± 2.9 (17)	− 1.0 ± 3.0 (6)	**− 2.0 ± 1.8 (10)**	0.392
PQAS 17—heavy	Mean ± SD (n)	− 1.1 ± 1.5 (16)	− 1.1 ± 2.6 (17)	− 0.2 ± 3.3 (6)	**− 2.2 ± 2.7 (10)**	0.418
PQAS 18—unpleasant	Mean ± SD (n)	**− 3.2 ± 3.1 (16)**	− 1.5 ± 2.0 (17)	− 1.7 ± 2.3 (6)	− 1.9 ± 2.3 (10)	0.265
PQAS 19a—intense deep	Mean ± SD (n)	**− 2.7 ± 3.4 (16)**	− 1.1 ± 2.7 (15)	− 0.7 ± 3.0 (6)	− 1.4 ± 2.4 (9)	0.385
PQAS 19b—intense surface	Mean ± SD (n)	**− 2.3 ± 2.5 (16)**	− 0.9 ± 2.3 (15)	− 0.7 ± 5.0 (6)	− 0.7 ± 3.1 (9)	0.476
PQAS 20—time quality (intermittent, variable, stable)	Mean ± SD (n)	0.1 ± 0.6 (15)	0.1 ± 0.4 (15)	− 0.6 ± 0.9 (5)	− 0.1 ± 0.6 (9)	0.090

Group 1: intervention (lower extremity, LE) massage 3 times per week for 4 weeks.

Group 2: intervention (lower extremity, LE) massage 2 times per week for 6 weeks.

Group 3: control (head/neck/shoulder, HNS) massage 3 times per week for 4 weeks.

Group 4: control (head/neck/shoulder, HNS) massage 2 times per week for 6 weeks.

Bold represents > 2spoint change (improvement).

Incorrect:

**Table 5:** Individual PQAS changes scores [End-of-Study (week 10) minus Baseline].

PQAS items	Group 1 LE	Group 2 LE	Group 3 HNS	Group 4 HNS	ANOVA	*p*-value
PQAS 1—intense	Mean ± SD (n)	**− 3.1 ± 2.8 (16)**	− 1.4 ± 1.8 (17)	− 0.8 ± 3.7 (6)	− 1.4 ± 2.2 (10)	0.126
PQAS 2—sharp	Mean ± SD (n)	**− 3.4 ± 3.2 (16)**	− 1.8 ± 2.6 (17)	− 0.3 ± 2.7 (6)	− 1.3 ± 2.3 (10)	0.088
PQAS 3—hot	Mean ± SD (n)	− 1.7 ± 2.9 (16)	− 1.4 ± 2.7 (17)	− 1.5 ± 1.8 (6)	**− 2.0 ± 1.9 (10)**	0.947
PQAS 4—dull	Mean ± SD (n)	**− 3.0 ± 3.5 (16)**	− 0.9 ± 3.3 (17)	0.2 ± 4.6 (6)	− 0.5 ± 3.0 (10)	0.152
PQAS 5—cold	Mean ± SD (n)	**− 2.2 ± 2.3 (16)**	− 0.8 ± 4.1 (17)	− 1.3 ± 1.8 (6)	− 0.2 ± 1.5 (9)	0.377
PQAS 6—sensitive	Mean ± SD (n)	**− 2.9 ± 2.7 (16)**	− 0.1 ± 3.2 (17)	0.0 ± 3.8 (6)	1.0 ± 2.4 (9)	0.010
PQAS 7—tender	Mean ± SD (n)	**− 3.1 ± 3.1 (16)**	− 1.4 ± 1.9 (17)	− 0.8 ± 3.5 (6)	− 0.3 ± 1.6 (10)	0.040
PQAS 8—itchy	Mean ± SD (n)	− 1.7 ± 2.4 (16)	− 0.1 ± 2.9 (17)	− 0.2 ± 1.0 (6)	− 0.4 ± 1.8 (10)	0.254
PQAS 9—shooting	Mean ± SD (n)	**− 2.9 ± 2.3 (16)**	− 1.6 ± 3.6 (17)	0.2 ± 2.0 (6)	− 0.8 ± 1.3 (10)	0.072
PQAS 10—numb	Mean ± SD (n)	**− 2.8 ± 3.0 (16)**	− 1.5 ± 2.9 (17)	**− 2.0 ± 2.4 (6)**	− 1.7 ± 2.0 (10)	0.549
PQAS 11—electrical	Mean ± SD (n)	**− 2.7 ± 2.5 (16)**	0.1 ± 3.8 (17)	0.3 ± 4.2 (6)	**− 2.0 ± 2.2 (10)**	0.057
PQAS 12—tingling	Mean ± SD (n)	**− 3.4 ± 2.7 (16)**	− 1.2 ± 2.1 (17)	− 1.8 ± 1.9 (6)	**− 2.1 ± 2.5 (10)**	0.081
PQAS 13—cramping	Mean ± SD (n)	**− 2.4 ± 2.3 (16)**	− 1.1 ± 3.1 (17)	**− 3.7 ± 3.0 (6)**	− 0.5 ± 2.1 (10)	0.076
PQAS 14—radiating	Mean ± SD (n)	**− 2.3 ± 2.8 (16)**	− 1.0 ± 2.9 (17)	1.7 ± 2.9 (6)	− 0.9 ± 1.9 (10)	0.033
PQAS 15—throbbing	Mean ± SD (n)	− 1.9 ± 2.6 (16)	− 1.2 ± 3.1 (16)	− 1.2 ± 2.7 (6)	− 0.4 ± 1.5 (10)	0.583
PQAS 16—aching	Mean ± SD (n)	**− 2.3 ± 3.1 (16)**	− 0.7 ± 2.9 (17)	− 1.0 ± 3.0 (6)	**− 2.0 ± 1.8 (10)**	0.392
PQAS 17—heavy	Mean ± SD (n)	− 1.1 ± 1.5 (16)	− 1.1 ± 2.6 (17)	− 0.2 ± 3.3 (6)	**− 2.2 ± 2.7 (10)**	0.418
PQAS 18—unpleasant	Mean ± SD (n)	**− 3.2 ± 3.1 (16)**	− 1.5 ± 2.0 (17)	− 1.7 ± 2.3 (6)	− 1.9 ± 2.3 (10)	0.265
PQAS 19a—intense deep	Mean ± SD (n)	**− 2.7 ± 3.4 (16)**	− 1.1 ± 2.7 (15)	− 0.7 ± 3.0 (6)	− 1.4 ± 2.4 (9)	0.385
PQAS 19b—intense surface	Mean ± SD (n)	−** 2.3 ± 2.5 (16)**	− 0.9 ± 2.3 (15)	− 0.7 ± 5.0 (6)	− 0.7 ± 3.1 (9)	0.476
PQAS 20—time quality (intermittent, variable, stable)	Mean ± SD (n)	0.1 ± 0.6 (15)	0.1 ± 0.4 (15)	− 0.6 ± 0.9 (5)	− 0.1 ± 0.6 (9)	0.090

Group 1: intervention (lower extremity, LE) massage 3 times per week for 4 weeks.

Group 2: intervention (lower extremity, LE) massage 2 times per week for 6 weeks.

Group 3: control (head/neck/shoulder, HNS) massage 3 times per week for 4 weeks.

Group 4: control (head/neck/shoulder, HNS) massage 2 times per week for 6 weeks.

Bold represents > 2-point change (improvement).

The original Article has been corrected.

